# Increased levels of ficolin-1 and of C3dg are independently associated with high risk of infection in patients with chronic kidney disease: a prospective cohort study

**DOI:** 10.3389/fimmu.2025.1645347

**Published:** 2025-10-01

**Authors:** Solbjørg Sagedal, Markus Hovd, Anders Åsberg, Tom Eirik Mollnes, Olav Klingenberg, Torbjørn Fossum Heldal, Bartlomiej J. Witczak, Trine Korsgaard Hejlesen, Anne Troldborg, Steffen Thiel

**Affiliations:** ^1^ Department of Nephrology, Oslo University Hospital Ullevål, Oslo, Norway; ^2^ Section of Nephrology, Department of Transplantation Medicine, Oslo University Hospital Rikshospitalet, Oslo, Norway; ^3^ Department of Pharmacy, University of Oslo, Oslo, Norway; ^4^ Department of Immunology, Oslo University Hospital Rikshospitalet and University of Oslo, Oslo, Norway; ^5^ Research Laboratory, Nordland Hospital, Bodø, Norway; ^6^ Department of Medical Biochemistry, Oslo University Hospital, Oslo, Norway; ^7^ Institute of Clinical Medicine, University of Oslo, Oslo, Norway; ^8^ Department of Nephrology, Akershus University Hospital, Lørenskog, Norway; ^9^ Department of Biomedicine, University of Aarhus, Aarhus, Denmark; ^10^ Department of Rheumatology, Aarhus University Hospital, Aarhus, Denmark

**Keywords:** chronic kidney disease, infection, complement, lectin pathway, C3dg, ficolin-1, pattern recognition molecules

## Abstract

**Background and hypothesis:**

Infection is a leading cause of morbidity and mortality in patients with chronic kidney disease (CKD). The complement system provides crucial first-line defense against pathogens. Mannose-binding lectin (MBL), ficolins (1, 2 and 3), and collectin-LK 1 (CL-LK) are pattern recognition molecules (PRMs) of the lectin pathway (LP) that recognize microbial surfaces and activate complement. C3dg is a complement cleavage fragment indicating complement activation. The aim of the study was to investigate whether levels of PRMs and C3dg are associated with the risk of significant infections requiring hospitalization in patients with CKD.

**Methods:**

This prospective cohort study included 518 patients ≥18 years with CKD (eGFR<60 mL/min/1.73 m^2^), consecutively recruited between 2008-2022. About half (270/518) were in dialysis at inclusion. None of the patients were previously transplanted with any organ or stem cells. The primary endpoint was non-access-related infections requiring hospitalization. Patients were followed until kidney transplantation or death or until 31.12.2024. Plasma concentrations of the biomarkers were measured at inclusion. Time-to-event analyses using Cox regression were employed to assess associations with infection, adjusting for age, sex, diabetes, dialysis status, and dialysis vintage.

**Results:**

During a median (interquartile range [IQR]) time of follow-up of 1.24 (0.49-2.76) years, 182 patients (35%) were hospitalized due to non-access infection. Higher baseline levels of ficolin-1 and C3dg were independently associated with infection risk, HR 3.05, 95% CI 1.25-7.43, p=0.01 and HR 2.97, 95% CI 1.37-6.44, p=0.006, respectively, for each log_10_ unit increase. In multivariable models including all biomarkers, only C3dg remained independently associated with infection (HR 2.81, 95% CI 1.23-6.43, p=0.01).

**Conclusions:**

High levels of complement activation (C3dg) and ficolin-1 were independently associated with increased infection risk in patients with CKD. A dysregulated complement activation rather than PRM deficiency seems to be a key pathogenic mechanism resulting in increased infection risk in advanced CKD.

## Introduction

Infection is the second leading cause of both hospitalization and mortality next to cardiovascular disease (CVD) in patients with chronic kidney disease (CKD), and this applies both to patients receiving hemodialysis (HD) or peritoneal dialysis (PD) as well as to those with non-dialysis-dependent CKD (1–5). CKD is associated with impairment of the adaptive immune system (6–9). As CKD progresses, this immunodeficiency may render patients more dependent on their innate immune defense mechanisms.

**Figure 1:**
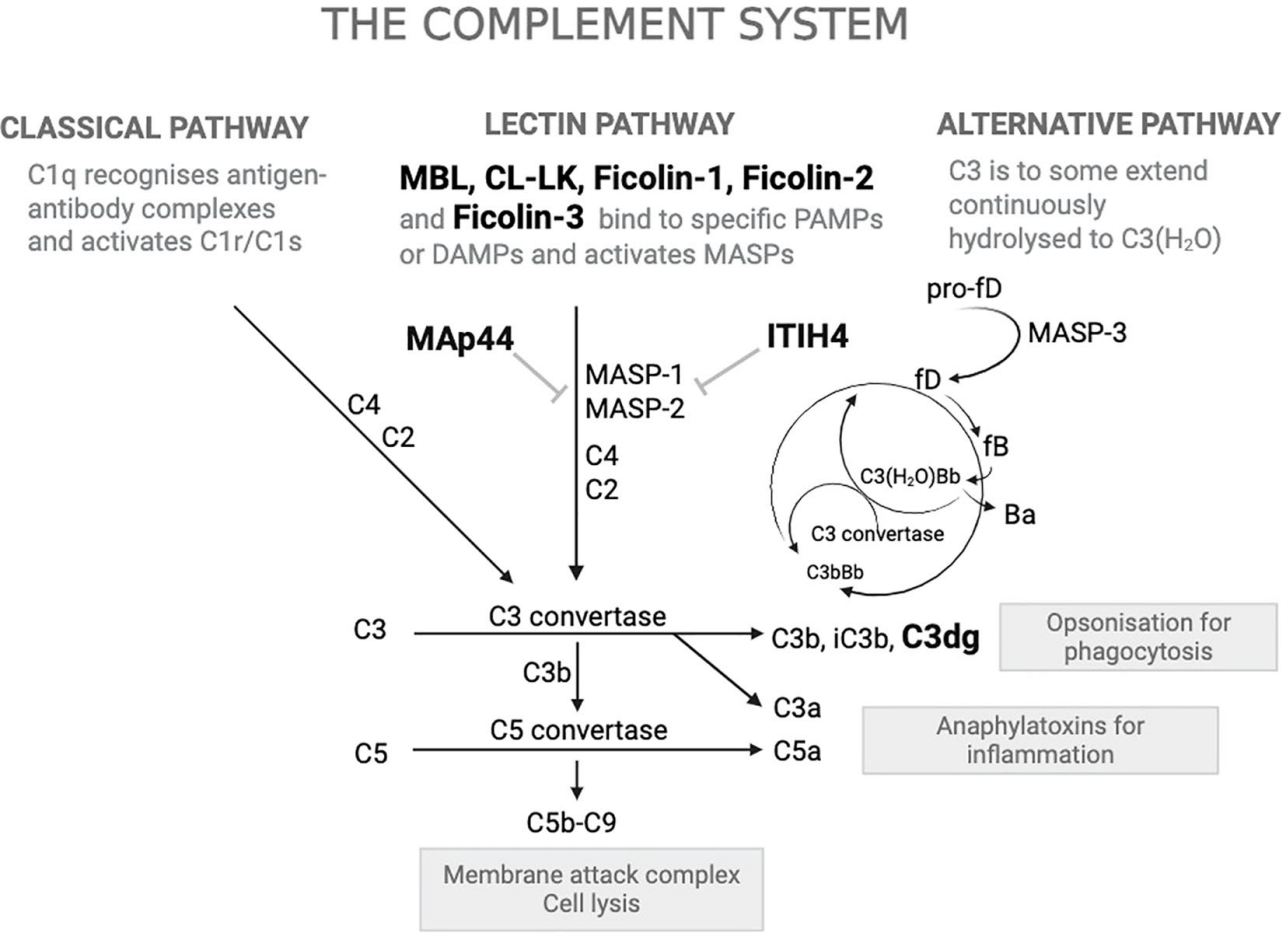
Overview of the complement system and the proteins quantified. The complement system is a crucial component of innate immunity, facilitating the clearance of pathogens and damaged cells via three activation pathways: the Classical Pathway, the Lectin Pathway, and the Alternative Pathway. Proteins highlighted in bold black (MBL, CL-LK, Ficolin-1, Ficolin-2, Ficolin-3, MAp44, ITIH4, C3dg) are those that have been quantified for this project. A flat-headed arrow indicates inhibition. PAMPs: Pathogen-Associated Molecular Patterns, DAMPs: Damage-Associated Molecular Patterns; MBL: Mannose-Binding Lectin; CL-LK: Collectin Liver-Kidney; MASPs: MBL-Associated Serine Proteases; fB: Factor B; fD: Factor D; Ba and Bb: Fragments of Factor B; MAC (C5b–C9): Membrane Attack Complex; MAp44: Mannan-binding lectin-associated protein of 44 kDa; ITIH4: Inter-alpha-trypsin inhibitor heavy chain 4.

The complement system (**Figure 1**) is a key component of the innate immune response, providing first-line defense against invading pathogens (10, 11). The lectin pathway (LP) is an integral component of this system. The LP is initiated when at least one of its pattern recognition molecules (PRMs) binds to specific molecular patterns on microbial surfaces or to altered host structures (12–14). The PRMs of the LP include the plasma proteins mannose-binding lectin (MBL, also named mannan-binding lectin), ficolin-1, ficolin-2, ficolin-3 (also named M-ficolin, L-ficolin and H-ficolin, respectively) and collectin-LK (CL-LK). Upon target recognition, these PRMs activate the complement cascade through their interaction with MBL-associated serine proteases (MASP-1, MASP-2, and MASP-3) (15–17). Among these PRMs, CL-LK is a heterotrimer composed of the two polypeptide chains CL-L1 and CL-K1, which circulate in plasma primarily in heteromeric form (18). In the present study, we measured CL-L1 as a surrogate for CL-LK since plasma levels of CL-L1 and CL-K1 are highly correlated (19).

Activation of the complement system leads to the generation of multiple cleavage fragments (**Figure 1**). In this study, we measured the plasma concentration of C3dg (often referred to as C3d), a fragment produced when complement factor C3 is cleaved (20). The presence of C3dg in plasma or deposited in tissues can serve as a marker for an activated complement cascade that has proceeded until the last cleavage steps of C3 and is considered a reliable marker of recent or ongoing complement activation (21).

In addition to PRMs and C3dg, we also measured MAp44, a 44 kDa MBL-associated protein that functions as a natural endogenous competitive inhibitor of LP activation, and ITIH4 (inter-alpha-trypsin inhibitor heavy chain H4), a recently identified inhibitor of the MASPs (22, 23). We hypothesized that baseline abnormalities in LP components or higher complement activation (as indicated by C3dg) would be associated with increased risk of clinically significant infections in CKD.

## Materials and methods

### Study design and population

Adult patients (≥18 years) with CKD, defined as an estimated glomerular filtration rate (eGFR) <60 mL/min/1.73 m^2^, and who were followed at Oslo University Hospital Ullevål, were consecutively recruited for this prospective observational study between 2008 and 2022. The protocol was approved by the Regional Ethics Committee (approval number S-08501d, 2008/14590), and written informed consent was obtained from all patients prior to inclusion according to the Helsinki Declaration.

Exclusion criteria were active malignant disease, previous transplantation with any organ or stem cells, active bacterial or virus infection, congenital or acquired immunodeficiency (including active hepatitis B or HIV infection), immunosuppressive therapy other than low-dose glucocorticoid (≤5 mg/day), drug abuse that may affect patient adherence, life expectancy less than one year and inability or unwillingness to provide informed consent.

### Response variables and endpoints

The primary outcome was a non-access-related, clinically significant infection, defined as an infection requiring hospitalization. Hospital-acquired infections were not recorded or adjusted for. Only infections that were the cause of admission, with symptoms prior to hospitalization, were included, minimizing risk of misclassification.

The primary outcome was registered from the date of study inclusion until the earliest of the following events: kidney transplantation, transfer to another hospital (loss to follow-up), death, or end of study (December 31, 2024). No events occurring after this date were recorded.

Patients who were not on dialysis at inclusion but initiated dialysis during follow-up were right censored at the date of dialysis initiation to avoid bias from dialysis-related factors. Thus, in this population infections occurring after the date of dialysis initiation were excluded from outcome registration. However, non-access-related infections that led directly to hospitalization before the date of dialysis initiation were included as events.

### Laboratory analyses

Blood samples were collected at inclusion. Blood samples were obtained immediately before the dialysis session for patients on HD at inclusion, either from the arteriovenous fistula or from the HD catheter. Samples were centrifuged at 2500 x g for 15 minutes at 4 °C, and plasma was frozen and stored at -80 °C until analysis. In-house developed time-resolved immunofluorometric assays (TRIFMAs) were applied to measure all proteins of the LP (except for ficolin-2, which was assessed using a commercially available enzyme-linked immunosorbent assay (ELISA) kit (Hycult Biotechnology, Uden, the Netherlands)). Detailed descriptions of the assays, including the applied custom-developed antibodies used in each assay, are provided elsewhere (24–27). Sample dilution and loading onto microtiter plates were automated using a pipetting robot (JANUS, PerkinElmer, Hamburg, Germany). All samples were analyzed in duplicates, and measurements were repeated if the coefficient of variation (CV) between duplicates exceeded 15%. Inter-assay CVs, determined using internal controls for each assay, were all below 15%. All laboratory analyses were performed blinded to clinical data, ensuring an unbiased approach.

### Estimation of sample size

1. The incidence of at least one infection requiring hospitalization in CKD stage 5 patients during one year of follow-up is approximately 40%, based on data from The Norwegian Renal Registry.
2. We hypothesized that patients with low levels of MBL and/or other PRMs or effector molecules of the LP would have an increased infection incidence of 60% within one year, representing a 20% absolute increase.
3. Based on previous studies in CKD stage 5 patients, the prevalence of low MBL and/or low levels of other PRMs or effector molecules was estimated at approximately 20% (28). This corresponds to a ratio of 4:1 between patients with high/normal versus low biomarker levels.

Using these assumptions, a total sample size of approximately 400 patients (80 with low biomarker levels and 320 with high/normal levels) would provide 90% power to detect the hypothesized difference, with a significance level of 5%.

Allowing for a 25% loss to follow-up or incomplete data, the final target sample size was set at 520 patients.

### Statistics

Time-to-event analyses were performed using Cox proportional hazards regression models to evaluate the association between plasma biomarker concentrations and time to first non-access-related infection requiring hospitalization. Analyses were conducted using a stepwise approach, beginning with univariable models for each biomarker separately. Subsequently, multivariable models were fitted adjusting for relevant clinical covariates, including age (continuous), dialysis vintage (continuous, log-transformed due to skewness), sex (male/female), diabetes at inclusion (yes/no), and dialysis treatment at inclusion (yes/no). Finally, a fully adjusted model was constructed including all measured biomarkers simultaneously. Biomarker concentrations were logarithmically transformed using the formula: y = log_10_(x + 0.0001) to account for non-normal distributions and to facilitate comparison of effect estimates. Assumptions of proportional hazards were evaluated using standard diagnostic methods, including visual inspection of Schoenfeld residuals in case of violations.

Patients not on dialysis at inclusion and who subsequently initiated dialysis during follow-up were right censored at the date of dialysis initiation. Statistical significance was defined as a two-sided p-value <0.05. All analyses were performed using R (version 4.4.2).

## Results

### Study population

Altogether 520 patients were included in the study: 378 (73%) men and 142 (27%) women. Two patients withdrew their informed consent, leaving 518 patients available for statistical analyses. The primary kidney diseases were hypertensive nephrosclerosis in 208 (40%), diabetic nephropathy in 148 (29%), chronic glomerulonephritis in 64 (12%), autosomal dominant polycystic kidney disease in 49 (9.5%), chronic tubulointerstitial disease in 15 (2.9%), kidney tumor in 4 (0.8%), AA amyloidosis in 2 (0.4%) and unknown in 28 (5.4%). Baseline characteristics of the study cohort are presented in **Table 1**. At inclusion in the study, 270 (52%) patients were on dialysis, 228 (84%) of them received HD and 42 (16%) received PD. The median (IQR) dialysis vintage prior to inclusion for these patients was 159 (62–428) days. The median (interquartile range [IQR]) eGFR in 248 patients not on dialysis at inclusion was 18 (13–27) mL/min/1.73 m^2^. During the study period, an additional 130 patients became dialysis dependent and initiated dialysis treatment, including 63 who started HD and 67 who started PD.

**Table 1:** Patient characteristics and biomarkers at time of inclusion.

Variable	N/p-concentration	Median/Part	25%-tile	75%-tile
sex				
Female	141	27%		
Male	377	73%		
Age (years)	518	65	55	73
Ethnicity				
African	37	7%		
Asian	63	12%		
Hispanic	2	<1%		
White	416	80%		
BMI (kg/m^2^)	516	26	23	30
eGFR (mL/min/1.73 m^2^)	490	11	7	18
Diabetes mellitus				
No	310	60%		
Yes	208	40%		
C3dg (mU/mL)	518	27	21	35
Ficolin-1 (ng/mL)	518	4214	3513	5222
MBL (ng/mL)	518	1880	563	3492
CL-L1 (ng/mL)	518	592	539	657
ITIH4 (µg/mL)	518	261	232	294
Ficolin-2 (ng/mL)	518	3927	2840	4808
Ficolin-3 (ng/mL)	518	26203	21373	30475
MAp44 (ng/mL)	518	2402	2034	2817
CRP (mg/L)	518	3.1	1.5	7.3

BMI, Body Mass Index; eGFR, estimated glomerular filtration rate; MBL, mannose binding lectin; MAp44, MBL-associated protein of 44 kDa; CRP, C-reactive protein.

### Infections

Patients not on dialysis at inclusion and who subsequently started dialysis (N = 130) during follow-up were censored at the date of dialysis initiation, and infections occurring after this point were not considered in the statistical analyses. That means that non-access-related infections requiring hospitalization were recorded in all the 518 patients, but in the 130 patients who were pre-dialytic at inclusion and who later started in dialysis, no infections occurring after the date of dialysis initiation were recorded. Non-access-related infections were recorded over a median (IQR) observation period of 1.24 (0.49- 2.76) years. During this time, 182 (35%) patients experienced at least one hospitalization due to non-access-related infection. In other words, 518 patients were included in the final analyses, and 182 of these experienced at least one non-access-related infection episode requiring hospitalization, and these 182 infection episodes were included as outcome events in the final statistical analyses. These infections were defined as any infection requiring hospitalization except infections related to dialysis access (HD or PD catheters, arteriovenous fistulas) or PD peritonitis.

Among the 182 patients with non-access-related infections, the median (range) number of hospitalizations was 2 (1-12). A total of 170 patients were hospitalized at least once due to bacterial infection, and 39 patients were hospitalized at least once due to viral infection.

The most frequent causes of infection-related hospitalization were lower respiratory tract infections or pneumonia (101 patients), urinary tract infections (50), skin infections (37), bacteraemia or septicaemia (29), intraabdominal infections or colitis (23), upper respiratory tract infections (18), osteomyelitis (12), gastroenteritis or esophagitis (18), arthritis (2), endocarditis (4), and glandular tuberculosis (1). In addition, 11 patients were hospitalized due to infections of unknown origin.

### Associations between clinical covariates and risk of infection

Univariable Cox regression analyses were performed to evaluate the association between clinical covariates at inclusion and the risk of non-access-related infections leading to hospitalization. Patient age, diabetes, dialysis at inclusion and dialysis vintage were all highly significantly associated with increased infection risk (**Supplementary Table S1**).

In a multivariable analysis of clinical covariates as risk factors of non-access-related infection requiring hospitalization, patient age and diabetes at inclusion remained highly significantly associated with infection risk (**Supplementary Table S2**).

### Associations between biomarkers and risk of infection

The biomarkers were analyzed at inclusion in all the 518 patients, and there were no missing values. That means that for each of the 8 biomarkers, 518 samples were included in the final statistical analyses. Univariable Cox regression analyses were performed to evaluate the association between plasma biomarkers measured at inclusion and the risk of non-access-related infections leading to hospitalization (**Table 2**). Higher levels of C3dg, CRP, ficolin-1 and CL-L1 were each statistically significantly associated with increased infection risk, with hazard ratios (HR) per log_10_ unit increase of 2.53 (95% CI 1.19-5.37, p=0.02), 1.29 (95% CI 1.04-1.59, p=0.02), 4.67 (95% CI 1.99-10.93, p<0.001) and 4.95 (95% CI 1.04-23.59, p=0.04), respectively. For C3dg, a violation of the assumption of proportional hazards (p=0.0146) was found. Visual inspection of scales Schoenfeld residuals revealed the violation to be limited in time, around 0.1 years (**Supplementary Table S3**, **Supplementary Figure S1**).

**Table 2:** Univariable analyses of biomarkers on first non-access-related infections requiring hospitalization.

Parameters at inclusion	Hazard ratio	95% CI	p-value
log10(C3dg) (mU/mL)	2.53	1.19-5.37	0.02
log10(Ficolin-1) (ng/mL)	4.67	1.99-10.93	<0.001
log10(MBL) (ng/mL)	1.03	0.87-1.21	0.7
log10(CL-L1) (ng/mL)	4.95	1.04-23.59	0.04
log10(Ficolin-3) (ng/mL)	0.78	0.50-1.21	0.3
log10(Ficolin-2) (ng/mL)	0.69	0.35-1.36	0.3
log10(ITIH4) (µg/mL)	2.65	0.46-15.11	0.3
log10(MAp44) (ng/mL)	0.38	0.11-1.32	0.1
log10(CRP) (mg/L)	1.29	1.04-1.59	0.02

Univariable Cox models regressing the individual biomarker on first non-access-related infections requiring hospitalization were used.

In multivariable Cox regression models adjusting for age, sex, diabetes at inclusion, dialysis treatment at inclusion, and dialysis vintage before inclusion, both C3dg and ficolin-1 remained independently associated with increased infection risk. The HRs per log_10_ unit increase were 2.97 (95% CI 1.37-6.44, p=0.006) for C3dg and 3.05 (95% CI 1.25-7.43, p=0.01) for ficolin-1, (**Tables 3**, **4**).

**Table 3:** Multivariable analysis of risk factors of non-access-related infections requiring hospitalization.

Parameters at inclusion	Hazard ratio	95% CI	p-value
log_10_ (C3dg) (mU/mL)	2.97	1.37-6.44	0.006
Age, years	1.02	1.01-1.03	<0.001
Sex, male	0.81	0.61-1.10	0.175
Diabetes	1.67	1.28-2.18	<0.001
Dialysis at inclusion	2.55	0.71-9.14	0.151
log_10_ (dialysis vintage, years)	0.94	0.67-1.33	0.746

A Cox model regressing time to first non-access-related infection requiring hospitalization on the log_10_-transformed biomarker C3dg, adjusted for age at time of inclusion, sex, dialysis at inclusion, diabetes at inclusion, and time in dialysis at time of inclusion in years was used.

**Table 4:** Multivariable analysis of risk factors of non-access-related infections requiring hospitalization.

Parameters at inclusion	Hazard ratio	95% CI	p-value
log_10_ (Ficolin-1) (ng/mL)	3.05	1.25-7.43	0.01
Age, years	1.02	1.01-1.03	<0.001
Sex, male	0.85	0.63-1.14	0.27
Diabetes	1.64	1.25-2.14	<0.001
Dialysis at inclusion	2.39	0.68-8.42	0.17
log_10_ (dialysis vintage, years)	0.99	0.69-1.35	0.83

A Cox model regressing time to first non-access-related infection requiring hospitalization on the log_10_-transformed biomarker ficolin-1, adjusted for age at time of inclusion, sex, dialysis at inclusion, diabetes at inclusion, and time in dialysis at time of inclusion in years was used.

For C3dg, the individual variable was found to violate the assumption of proportional hazards (P = 0.04), but the global test for proportional hazards detected no influence on the full model (p=0.099, **Supplementary Table S4**).

In a comprehensive multivariable Cox regression model, including all measured biomarkers (MBL, ficolin-1, ficolin-2, ficolin-3, CL-L1, C3dg, MAp44, and ITIH4), alongside the previously mentioned covariates, only C3dg remained significantly associated with increased infection risk, (HR 2.81, 95% CI: 1.23-6.43, p=0.01). In this model, patient age and diabetes mellitus at inclusion were also independently associated with infection risk (**Table 5**). To assess potential collinearity, we calculated and have plotted variance inflation factors (VIFs) for the full model (**Supplementary Figure S2**). Notably, VIFs were >5 for the clinical covariates dialysis at inclusion and dialysis vintage. In a reduced model excluding these covariates, no VIFs were >2.

**Table 5:** Multivariable analysis of risk factors of non-access-related infections requiring hospitalization.

Parameters at inclusion	Hazard ratio	95% CI	p-value
Age, years	1.02	1.01-1.03	<0.001
Sex, male	0.85	0.62-1.16	0.30
Diabetes	1.65	1.25-2.17	<0.001
Dialysis at inclusion	2.32	0.64-8.48	0.2
log10 (dialysis vintage, years)	0.97	0.69-1.37	0.86
log10 (MBL) (ng/mL)	1.06	0.90-1.25	0.46
log10 (Ficolin-1) (ng/mL)	2.44	0.95-6.26	0.06
log10 (Ficolin-2) (ng/mL)	1.38	0.68-2.80	0.37
log10 (Ficolin-3) (ng/mL)	0.80	0.53-1.20	0.28
log10 (CL-L1) (ng/mL)	2.19	0.33-14.34	0.41
log10 (C3dg) (mU/mL)	2.81	1.23-6.43	0.01
log10 (MAp44) (ng/mL)	0.74	0.21-2.67	0.65
log10 (ITIH4) (µg/mL)	1.02	0.15-7.09	0.99

A Cox model regressing time to first non-access-related infection requiring hospitalization on the log10-transformed biomarkers adjusted for age at time of inclusion, sex, dialysis at inclusion, diabetes at inclusion, and time in dialysis at time of inclusion in years was used.

For all models, the assumption of proportional hazards was evaluated, and are available in the **Supplementary Materials**, (**Supplementary Tables S3**-**S6**).

Baseline biomarker concentrations in patients with and without non-access-related infections are presented in **Table 6**. Baseline plasma concentrations of C3dg, CRP, Ficolin-1 and MAp44 were the same in patients with and without dialysis at the time of inclusion (**Supplementary Table S7**).

**Table 6:** Baseline biomarker plasma concentrations in patients with and without non-access-related infections. Data presented as median (IQR).

Parameters at inclusion	Patients with at least one non-access-related infection (N = 182)	Patients with no non-access-related infection (N = 336)
C3dg mU/mL	29 (21-38)	27 (20-34)
CRP mg/L	4.4 (2.0-11.0)	2.6 (1.3-5.5)
Ficolin-1 ng/mL	4460 (3615-5567)	4124 (3450-4124)
MAp44 ng/mL	2275 (1948-2641)	2442 (2075-2885)

We also performed a sensitivity analysis restricted to patients who remained pre-dialytic throughout follow-up, (**Supplementary Table S8**). Results did not show consistent associations between C3dg and infection risk in this subgroup of predialytic patients. However, according to our power analysis we needed to include 520 patients to detect the hypothesized difference, allowing for a 25% loss to follow-up. Therefore, the lack of a statistically significant association between C3dg and infection outcome in this subgroup may be due to a lack of power.

## Discussion

### Key findings of the study

In this prospective, extensive study of patients with CKD, we found that higher plasma concentrations of C3dg and ficolin-1 at baseline were independently associated with an increased risk of non-access-related infections requiring hospitalization. Importantly, in a fully adjusted multivariable model including all measured LP proteins and regulators and C3dg, only C3dg remained independently associated with infection risk, underscoring the potential importance of systemic complement activation rather than PRM concentration per se in infection susceptibility.

### Prior research on MBL deficiency

Previous studies investigating the role of the LP components in infection risk have focused predominantly on MBL deficiency, as MBL was the first recognized PRM within this pathway. Findings from population-based studies have been inconsistent. For example, an extensive study of over 9000 Caucasians found no association between MBL deficiency (defined genetically) and increased susceptibility to infection (29). In contrast, MBL deficiency has been linked to an increased risk of fatal pneumococcus infection (30), severe infections following chemotherapy or transplantation (31, 32), and recurrent respiratory tract infections in children (33).

However, evidence from dialysis populations is less supportive of MBL deficiency being a significant risk factor. In a study of 244 HD patients, no association between low MBL levels and life-threatening infections was found, possibly due to compensatory increases in other PRMs such as ficolin-2 (34).

### Novelty of the study

In our study, neither MBL nor other PRMs nor the regulatory proteins MAp44 or ITIH4 were associated with infection risk after adjustment for relevant clinical covariates and all measured biomarkers. Instead, C3dg, a cleavage product generated during complement activation, emerged as the most consistent and robust predictor of infection.

The aim of the present study was to investigate whether abnormalities in the LP could influence the incidence of infection in a cohort of patients with CKD. High levels of ficolin-1 and high levels of C3dg were independently associated with an increased risk of non-access-related infection. This contrasts with the traditional view that PRMs, including ficolin-1, protect against infectious disease, and the results deviate from previous reports that focus on deficiencies in the LP as a risk factor for infections. However, the pathological mechanisms are not fully elucidated. One may speculate whether ficolin-1 may be adsorbed to the polysulfone dialysis membrane, leading to a transient decrease in the plasma level of ficolin-1, thereby increasing the risk of infection. However, this mechanism could apply only to HD patients in this study, and other mechanisms are involved.

### C3dg and complement activation

Elevated levels of C3dg are widely recognized as a marker of recent or ongoing complement activation (21). C3dg may not only reflect systemic inflammation but may also contribute directly to immune modulation. Notably, C3dg can bind complement receptor 2 (CR2, CD21) on B cells, having the result that cytokine production, proliferation and antibody generation were all inhibited (35). This immunomodulatory role may partly explain why higher circulating C3dg levels could predispose to infection, particularly in a population with impaired adaptive immunity, such as patients with CKD. An alternative explanation for the association between elevated C3dg and increased infection risk may lie in the concept of complement exhaustion. Thus, rather than reflecting robust immune defense, elevated C3dg in CKD may indicate a fatigued complement system unable to respond adequately when challenged.

Complement activation in dialysis settings, both HD and PD, has been well documented (38, 39, 42–44). However, these studies have mainly explored immediate or local complement activation rather than long-term infection risk. In our cohort, about half the patients were not in dialysis at inclusion. Patients who subsequently initiated dialysis were censored at dialysis initiation, and thus, our findings may likely reflect mechanisms beyond dialysis-specific complement activation.

Interestingly, several studies have demonstrated that complement dysregulation may precede dialysis and may already be active in earlier stages of CKD. High MBL levels have been associated with the progression of diabetic nephropathy and complement activation has been implicated in kidney injury in both type I and type 2 diabetes (45–47). In line with this, nearly half of our study population was not on dialysis at inclusion. Yet, associations between complement activation markers and infection risk were found in the complete study population. This supports the hypothesis that patients with advanced CKD, irrespective of dialysis, may rely more heavily on complement-mediated defenses, as adaptive immunity is often impaired (6–8).

### Ficolin-1

Ficolin-1, although traditionally considered a protective PRM, was also independently associated with increased infection risk in our adjusted models (except when controlling for C3dg). The biological explanation remains unclear. Ficolin-1 is found in secretory granules in monocytes, neutrophils and type II alveolar epithelial cells in lungs and is secreted upon stimulation and on the basis of RNA-sequencing data cytokines have been shown to influence the expression from the FCN1 gene (36, 37). Its elevation could reflect systemic inflammation, immune activation, or increased turnover or consumption. Unlike ficolin-2, which is adsorbed to dialysis membranes, data on ficolin-1 behavior on dialysis are scarce (38, 39). While membrane adsorption might contribute, this mechanism cannot explain the association in non-dialysis patients in our study.

### MAp44 and CL-L1

In the present study, no association between MAp44 and infection risk could be demonstrated. In a previous study of kidney transplant recipients, low levels of MAp44 measured in patients with CKD immediately before kidney transplantation were found to be independently associated with increased mortality due to infectious disease (40). Furthermore, lower levels of MAp44 were found in patients with common variable immunodeficiency compared to healthy individuals (41). These findings may indicate a role for MAp44 in microbial defense, but its role in CKD remains to be thoroughly investigated.

In a univariable model, CL-L1 was significantly associated with infection risk, albeit with a wide confidence interval. However, this association was no longer evident when adjusting for relevant clinical covariates.

### Strengths of the study

The main strengths of this study include the relatively large, prospectively recruited cohort of patients with CKD, the comprehensive measurement of LP components and regulators and complement activation, and the systematic and rigorous registration of clinically significant, non-access-related infections requiring hospitalization.

### Study limitations

However, the study has limitations. First, the lack of a healthy control group prevents direct comparison of biomarker levels to those of non-CKD populations. Second, biomarkers were measured at a single time point, and their levels might vary over time or in response to intercurrent events. Third, while we adjusted for several key confounders, residual confounding by unmeasured factors (e.g., inflammation, nutrition, or medication use) cannot be excluded. Furthermore, the inclusion of both dialysis patients and patients not on dialysis resulted in a heterogenous study population, and this may represent a limitation of the study. Despite adjusting for dialysis at inclusion and dialysis vintage before inclusion, the different stages of kidney disease may have influenced the infection risk. However, 75% of patients not on dialysis at inclusion were CKD stage 4 or 5, confirming that almost all the patients in the study had a significant degree of kidney disease. Finally, the observational design precludes causal inference.

## Conclusions

In conclusion, this prospective study demonstrates that higher plasma levels of C3dg and ficolin-1 are associated with an increased risk of significant infections in patients with CKD. The association with C3dg persisted even after adjusting for all other LP components, highlighting the potential clinical relevance of systemic complement activation in this population. However, C3dg levels were only modestly higher in those who developed infections. This suggests C3dg may be marking a state of low-grade chronic inflammation that predisposes patients with CKD to infection. In fact, the modest median differences between groups suggest that these biomarkers may be more useful for population-level risk assessment than for precise individual patient prediction. A main conclusion is that the independent association of C3dg with increased infection risk may contribute to a shift in thinking from PRM deficiency toward a dysregulated complement activation as a risk factor in advanced CKD. The results must be interpreted with caution until confirmed in future even larger studies, which are warranted to explore the mechanisms underlying these associations and to investigate whether modulation of complement activation could represent a therapeutic target in infection-prone patients with CKD.

## Data Availability

The datasets presented in this study can be found in online repositories. The names of the repository/repositories and accession number(s) can be found below: De-identified data may be shared upon reasonable request and after application to the regional Committee for medical and health research Ethics (REK Sørøst, Norway), in cooperation with the authors. requests to access the datasets should be directed to SS, email: uxsasc@ous-hf.no.

## References

[1] James MT Quan H Tonelli M Manns BJ Faris P Laupland KB. CKD and risk of hospitalization and death with pneumonia. Am J Kidney Dis. (2009) 54:24–32. doi: 10.1053/j.ajkd.2009.04.005, PMID: 19447535

[2] Wang HE Gamboa C Warnock DG Muntner P. Chronic kidney disease and risk of death from infection. Am J Nephrol. (2011) 34:330–6. doi: 10.1159/000330673, PMID: 21860228 PMC3169360

[3] Johansen KL Gilbertson DT Li S Li S Liu J Roetker NS. US renal data system 2023 annual data report: epidemiology of kidney disease in the United States. Am J Kidney Dis. (2024) 83:A8–A13. doi: 10.1053/j.ajkd.2024.01.001, PMID: 38519262

[4] Sarnak MJ Jaber BL. Pulmonary infectious mortality among patients with end-stage renal disease. Chest. (2001) 120:1883–7. doi: 10.1378/chest.120.6.1883, PMID: 11742917

[5] Slinin Y Foley RN Collins AJ. Clinical epidemiology of pneumonia in hemodialysis patients: the USRDS waves 1, 3, and 4 study. Kidney Int. (2006) 70:1135–41. doi: 10.1038/sj.ki.5001714, PMID: 16871243

[6] Meuer SC Hauer M Kurz P Meyer zum Buschenfelde KH Kohler H. Selective blockade of the antigen-receptor-mediated pathway of T cell activation in patients with impaired primary immune responses. J Clin Inves. (1987) 80:743–9. doi: 10.1172/JCI113129, PMID: 3497950 PMC442298

[7] Girndt M Sester M Sester U Kaul H Kohler H. Defective expression of B7-2 (CD86) on monocytes of dialysis patients correlates to the uremia-associated immune defect. Kidney Int. (2001) 59:1382–9. doi: 10.1046/j.1523-1755.2001.0590041382.x, PMID: 11260399

[8] Girndt M Kohler H Schiedhelm-Weick E Meyer zum Buschenfelde KH Fleischer B. T cell activation defect in hemodialysis patients: evidence for a role of the B7/CD28 pathway. Kidney Int. (1993) 44:359–65. doi: 10.1038/ki.1993.252, PMID: 7690861

[9] Dumann H Meuer S Meyer zum Buschenfelde KH Kohler H. Hepatitis B vaccination and interleukin 2 receptor expression in chronic renal failure. Kidney Int. (1990) 38:1164–8. doi: 10.1038/ki.1990.328, PMID: 2150086

[10] Mastellos DC Hajishengallis G Lambris JD. A guide to complement biology, pathology and therapeutic opportunity. Nat Rev Immunol. (2024) 24:118–41. doi: 10.1038/s41577-023-00926-1, PMID: 37670180

[11] Merle NS Church SE Fremeaux-Bacchi V Roumenina LT. Complement system part I - molecular mechanisms of activation and regulation. Front Immunol. (2015) 6:262. doi: 10.3389/fimmu.2015.00262, PMID: 26082779 PMC4451739

[12] Thiel S. Complement activating soluble pattern recognition molecules with collagen-like regions, mannan-binding lectin, ficolins and associated proteins. Mol Immunol. (2007) 44:3875–88. doi: 10.1016/j.molimm.2007.06.005, PMID: 17768106

[13] Degn SE Thiel S. Humoral pattern recognition and the complement system. Scand J Immunol. (2013) 78:181–93. doi: 10.1111/sji.12070, PMID: 23672641

[14] Dobo J Kocsis A Farkas B Demeter F Cervenak L Gal P. The lectin pathway of the complement system-activation, regulation, disease connections and interplay with other (Proteolytic) systems. Int J Mol Sci. (2024) 25:1566–602. doi: 10.3390/ijms25031566, PMID: 38338844 PMC10855846

[15] Sorensen R Thiel S Jensenius JC. Mannan-binding-lectin-associated serine proteases, characteristics and disease associations. Springer Semin Immunopathol. (2005) 27:299–319. doi: 10.1007/s00281-005-0006-z, PMID: 16189649

[16] Degn SE Jensen L Gal P Dobo J Holmvad SH Jensenius JC. Biological variations of MASP-3 and MAp44, two splice products of the MASP1 gene involved in regulation of the complement system. J Immunol Methods. (2010) 361:37–50. doi: 10.1016/j.jim.2010.07.006, PMID: 20673767

[17] Thiel S Vorup-Jensen T Stover CM Schwaeble W Laursen SB Poulsen K. A second serine protease associated with mannan-binding lectin that activates complement. Nature. (1997) 386:506–10. doi: 10.1038/386506a0, PMID: 9087411

[18] Henriksen ML Brandt J Andrieu JP Nielsen C Jensen PH Holmskov U. Heteromeric complexes of native collectin kidney 1 and collectin liver 1 are found in the circulation with MASPs and activate the complement system. J Immunol. (2013) 191:6117–27. doi: 10.4049/jimmunol.1302121, PMID: 24174618

[19] Troldborg A Hansen A Hansen SW Jensenius JC Stengaard-Pedersen K Thiel S. Lectin complement pathway proteins in healthy individuals. Clin Exp Immunol. (2017) 188:138–47. doi: 10.1111/cei.12909, PMID: 27925159 PMC5343365

[20] Zarantonello A Revel M Grunenwald A Roumenina LT. C3-dependent effector functions of complement. Immunol Rev. (2023) 313:120–38. doi: 10.1111/imr.13147, PMID: 36271889 PMC10092904

[21] Troldborg A Halkjaer L Pedersen H Hansen A Loft AG Lindegaard H. Complement activation in human autoimmune diseases and mouse models; employing a sandwich immunoassay specific for C3dg. J Immunol Methods. (2020) 486:112866. doi: 10.1016/j.jim.2020.112866, PMID: 32941885

[22] Degn SE Jensen L Olszowski T Jensenius JC Thiel S. Co-complexes of MASP-1 and MASP-2 associated with the soluble pattern-recognition molecules drive lectin pathway activation in a manner inhibitable by MAp44. J Immunol. (2013) 191:1334–45. doi: 10.4049/jimmunol.1300780, PMID: 23785123

[23] Pihl R Jensen RK Poulsen EC Jensen L Hansen AG Thogersen IB. ITIH4 acts as a protease inhibitor by a novel inhibitory mechanism. Sci Adv. (2021) 7. doi: 10.1126/sciadv.aba7381, PMID: 33523981 PMC7793589

[24] Krarup A Sorensen UB Matsushita M Jensenius JC Thiel S. Effect of capsulation of opportunistic pathogenic bacteria on binding of the pattern recognition molecules mannan-binding lectin, L-ficolin, and H-ficolin. Infect Immun. (2005) 73:1052–60. doi: 10.1128/IAI.73.2.1052-1060.2005, PMID: 15664949 PMC547010

[25] Thiel S Moller-Kristensen M Jensen L Jensenius JC. Assays for the functional activity of the mannan-binding lectin pathway of complement activation. Immunobiology. (2002) 205:446–54. doi: 10.1078/0171-2985-00145, PMID: 12396006

[26] Wittenborn T Thiel S Jensen L Nielsen HJ Jensenius JC. Characteristics and biological variations of M-ficolin, a pattern recognition molecule, in plasma. J Innate Immun. (2010) 2:167–80. doi: 10.1159/000218324, PMID: 20375634

[27] Axelgaard E Jensen L Dyrlund TF Nielsen HJ Enghild JJ Thiel S. Investigations on collectin liver 1. J Biol Chem. (2013) 288:23407–20. doi: 10.1074/jbc.M113.492603, PMID: 23814060 PMC3743509

[28] Sagedal S Thiel S Hansen TK Mollnes TE Rollag H Hartmann A. Impact of the complement lectin pathway on cytomegalovirus disease early after kidney transplantation. Nephrol Dial Transplant. (2008) 23:4054–60. doi: 10.1093/ndt/gfn355, PMID: 18577532

[29] Dahl M Tybjaerg-Hansen A Schnohr P Nordestgaard BG. A population-based study of morbidity and mortality in mannose-binding lectin deficiency. J Exp Med. (2004) 199:1391–9. doi: 10.1084/jem.20040111, PMID: 15148337 PMC2211811

[30] Eisen DP Dean MM Boermeester MA Fidler KJ Gordon AC Kronborg G. Low serum mannose-binding lectin level increases the risk of death due to pneumococcal infection. Clin Infect Dis. (2008) 47:510–6. doi: 10.1086/590006, PMID: 18611155 PMC7107952

[31] Peterslund NA Koch C Jensenius JC Thiel S. Association between deficiency of mannose-binding lectin and severe infections after chemotherapy. Lancet. (2001) 358:637–8. doi: 10.1016/S0140-6736(01)05785-3, PMID: 11530153

[32] Verschuren JJ Roos A Schaapherder AF Mallat MJ Daha MR de Fijter JW. Infectious complications after simultaneous pancreas-kidney transplantation: a role for the lectin pathway of complement activation. Transplantation. (2008) 85:75–80. doi: 10.1097/01.tp.0000297249.10654.f5, PMID: 18192915

[33] Cedzynski M Szemraj J Swierzko AS Bak-Romaniszyn L Banasik M Zeman K. Mannan-binding lectin insufficiency in children with recurrent infections of the respiratory system. Clin Exp Immunol. (2004) 136:304–11. doi: 10.1111/j.1365-2249.2004.02453.x, PMID: 15086395 PMC1809017

[34] Ishii M Ohsawa I Inoshita H Kusaba G Onda K Wakabayashi M. Serum concentration of complement components of the lectin pathway in maintenance hemodialysis patients, and relatively higher levels of L-Ficolin and MASP-2 in Mannose-binding lectin deficiency. Ther Apher Dial. (2011) 15:441–7. doi: 10.1111/j.1744-9987.2011.00936.x, PMID: 21974696

[35] Kovacs KG Macsik-Valent B Matko J Bajtay Z Erdei A. Revisiting the coreceptor function of complement receptor type 2 (CR2, CD21); coengagement with the B-cell receptor inhibits the activation, proliferation, and antibody production of human B cells. Front Immunol. (2021) 12:620427. doi: 10.3389/fimmu.2021.620427, PMID: 33868238 PMC8047317

[36] Liu Y Endo Y Iwaki D Nakata M Matsushita M Wada I. Human M-ficolin is a secretory protein that activates the lectin complement pathway. J Immunol. (2005) 175:3150–6. doi: 10.4049/jimmunol.175.5.3150, PMID: 16116205

[37] Chen X Gao Y Xie J Hua H Pan C Huang J. Identification of FCN1 as a novel macrophage infiltration-associated biomarker for diagnosis of pediatric inflammatory bowel diseases. J Transl Med. (2023) 21:203. doi: 10.1186/s12967-023-04038-1, PMID: 36932401 PMC10022188

[38] Mares J Thongboonkerd V Tuma Z Moravec J Matejovic M. Specific adsorption of some complement activation proteins to polysulfone dialysis membranes during hemodialysis. Kidney Int. (2009) 76:404–13. doi: 10.1038/ki.2009.138, PMID: 19421191

[39] Mares J Richtrova P Hricinova A Tuma Z Moravec J Lysak D. Proteomic profiling of blood-dialyzer interactome reveals involvement of lectin complement pathway in hemodialysis-induced inflammatory response. Proteomics Clin Appl. (2010) 4:829–38. doi: 10.1002/prca.201000031, PMID: 21137026

[40] Smedbraten J Mjoen G Hartmann A Asberg A Rollag H Mollnes TE. Low level of MAp44, an inhibitor of the lectin complement pathway, and long-term graft and patient survival; a cohort study of 382 kidney recipients. BMC Nephrol. (2016) 17:148. doi: 10.1186/s12882-016-0373-9, PMID: 27760523 PMC5070230

[41] Mistegaard CE Jensen L Christiansen M Bjerre M Jensen JMB Thiel S. Low levels of the innate immune system proteins MASP-2 and MAp44 in patients with common variable immunodeficiency. Scand J Immunol. (2022) 96:e13196. doi: 10.1111/sji.13196, PMID: 35673952 PMC9542173

[42] Nilsson B Ekdahl KN Mollnes TE Lambris JD. The role of complement in biomaterial-induced inflammation. Mol Immunol. (2007) 44:82–94. doi: 10.1016/j.molimm.2006.06.020, PMID: 16905192

[43] Young GA Kendall S Brownjohn AM. Complement activation during CAPD. Nephrol Dial Transplant. (1993) 8:1372–5. doi: 10.1093/ndt/8.12.1372, PMID: 8159307

[44] Sei Y Mizuno M Suzuki Y Imai M Higashide K Harris CL. Expression of membrane complement regulators, CD46, CD55 and CD59, in mesothelial cells of patients on peritoneal dialysis therapy. Mol Immunol. (2015) 65:302–9. doi: 10.1016/j.molimm.2015.02.005, PMID: 25725314

[45] Hansen TK Tarnow L Thiel S Steffensen R Stehouwer CD Schalkwijk CG. Association between mannose-binding lectin and vascular complications in type 1 diabetes. Diabetes. (2004) 53:1570–6. doi: 10.2337/diabetes.53.6.1570, PMID: 15161763

[46] Guan LZ Tong Q Xu J. Elevated serum levels of mannose-binding lectin and diabetic nephropathy in type 2 diabetes. PloS One. (2015) 10:e0119699. doi: 10.1371/journal.pone.0119699, PMID: 25803807 PMC4372410

[47] Bus P Chua JS Klessens CQF Zandbergen M Wolterbeek R van Kooten C. Complement activation in patients with diabetic nephropathy. Kidney Int Rep. (2018) 3:302–13. doi: 10.1016/j.ekir.2017.10.005, PMID: 29725633 PMC5932121

