# Supplementary material for: Increased levels of ficolin-1 and of C3dg are independently associated with high risk of infection in patients with chronic kidney disease: a prospective cohort study
Source: Front Immunol. 2025 Oct 1;16:1645347. doi: 10.3389/fimmu.2025.1645347 (PMC12521454; doi:10.3389/fimmu.2025.1645347)

Supplementary Materials

Increased Levels of Ficolin-1 and of C3dg are independently associated with high Risk of Infection in Patients with Chronic Kidney Disease: A Prospective Cohort Study

Sagedal Solbjorg[1*], Hovd Markus[2,3], Åsberg Anders[2,3], Mollnes Tom Eirik[4,5], Klingenberg Olav[6,7], Heldal Torbjørn[1,2], Witczak Bartlomiej[8], Hejlesen Trine Korsgaard[9], Troldborg Anne[9,10], Thiel Steffen[9].

[1]Department of Nephrology, Oslo University Hospital Ullevål, Oslo, [2]Section of Nephrology, Department of Transplantation Medicine, Oslo University Hospital Rikshospitalet, Oslo, [3]Department of Pharmacy, University of Oslo, [4]Department of Immunology, Oslo University Hospital Rikshospitalet and University of Oslo, [5]Research Laboratory, Nordland Hospital, Bodø, [6]Department of Medical Biochemistry, Oslo University Hospital, Oslo, [7]Institute of Clinical Medicine, University of Oslo, [8]Department of Nephrology, Akershus University Hospital, Lørenskog, Norway; and [9]Department of Biomedicine, University of Aarhus, Aarhus, Denmark, [10]Department of Rheumatology, Aarhus University Hospital, Denmark.

Running head: Complement and infection in CKD

Key words: chronic kidney disease, infection, complement, lectin pathway, C3dg, ficolin-1, pattern recognition molecules

Number of Supplementary Figures: 2

Number of Supplementary Tables: 8

Table S1: Univariable analyses of risk factors (clinical covariates only) of non-access-related infections requiring hospitalization.

	Model estimates			Proportionality assumptions	
Parameters at inclusion	Hazard ratio	95% CI	p-value	χ^2	p-value
Age, years	1.03	1.02-1.04	<0.001	0.188	0.665
Sex, male	0.79	0.59-1.05	0.100	0.985	0.321
Diabetes	1.68	1.29-2.19	<0.001	3.336	0.068
Dialysis at inclusion	2.28	1.72-3.01	<0.001	1.374	0.241
log10 (dialysis vintage, years)	1.23	1.14-1.33	<0.001	2.049	0.152

Table S2: Multivariable analysis of risk factors (clinical covariates only) of non-access-related infections requiring hospitalization.

	Model estimates			Proportionality assumptions	
Parameters at inclusion	Hazard ratio	95% CI	p-value	χ^2	p-value
Age, years	1.02	1.01-1.03	<0.001	0	1
Sex, male	0.84	0.62-1.12	0.23	1.013	0.314
Diabetes	1.62	1.24-2.12	<0.001	2.479	0.115
Dialysis at inclusion	2.95	0.83-10.47	0.09	0.929	0.335
log10 (dialysis vintage, years)	0.91	0.65-1.28	0.60	1.492	0.222
Global				6.109	0.296

Table S3: Proportional hazards assumptions for the univariable analyses of biomarkers on first non-access-related infections requiring hospitalization.

Parameters at inclusion	χ^2	p-value
log10(C3dg) (mU/mL)	5.961	0.015
log10(Ficolin-1) (ng/mL)	1.186	0.276
log10(MBL) (ng/mL)	1.111	0.292
log10(CL-L1) (ng/mL)	1.813	0.178
log10(Ficolin-3) (ng/mL)	0.746	0.388
log10(Ficolin-2) (ng/mL)	0.890	0.345
log10(ITIH4) (µg/mL)	0.016	0.898
log10(MAp44) (ng/mL)	1.334	0.248
log10(CRP) (mg/L)	0.033	0.855

See Table 2 for hazard ratios.

Table S4: Proportional hazards assumptions for the multivariable analysis of risk factors of non-access-related infections requiring hospitalization.

Parameters at inclusion	χ^2	p-value
$\log_{10}$ (C3dg) (mU/mL)	4.234	0.04
Age, years	0.055	0.814
Sex, male	1.009	0.315
Diabetes	2.318	0.128
Dialysis at inclusion	0.941	0.332
$\log_{10}$ (dialysis vintage, years)	1.466	0.226
Global	10.671	0.099

See Table 3 for hazard ratios.

Table S5: Proportional hazards assumptions for the multivariable analysis of risk factors of non-access-related infections requiring hospitalization.

Parameters at inclusion	χ^2	p-value
$\log_{10}$ (Ficolin-1) (ng/mL)	1.624	0.203
Age, years	0.005	0.945
Sex, male	0.93	0.335
Diabetes	2.459	0.117
Dialysis at inclusion	0.898	0.343
$\log_{10}$ (dialysis vintage, years)	1.434	0.231
Global	7.532	0.274

See Table 4 for hazard ratios.

Table S6: Proportional hazards assumptions for the multivariable analysis of risk factors of non-access-related infections requiring hospitalization.

Parameters at inclusion	χ^2	p-value
Age, years	0.156	0.693
Sex, male	0.865	0.352
Diabetes	2.144	0.143
Dialysis at inclusion	0.94	0.332
log10 (dialysis vintage, years)	1.471	0.225
log10 (MBL) (ng/mL)	0.557	0.456
log10 (Ficolin-1) (ng/mL)	0.89	0.345
log10 (Ficolin-2) (ng/mL)	0.063	0.802
log10 (Ficolin-3) (ng/mL)	1.259	0.262
log10 (CL-L1) (ng/mL)	1.76	0.185
log10 (C3dg) (mU/mL)	3.502	0.061
log10 (MAp44) (ng/mL)	1.02	0.312
log10 (ITIH4) (µg/mL)	0.488	0.482
Global	16.61	0.218

See Table 5 for hazard ratios.

Table S7: Baseline biomarker plasma concentrations in patients with and without dialysis at time of inclusion.

Parameters at inclusion	Patients with dialysis at inclusion (N=270)	Patients without dialysis at inclusion (N=248)
C3dg mU/mL	27 (21-35)	27 (21-34)
CRP mg/L	3.7 (1.7-8.8)	2.6 (1.3-5.1)
Ficolin-1 ng/mL	4242 (3480-5260)	4196 (3534-5184)
MAp44 ng/mL	2258 (1925-2663)	2520 (2136-2921)

Table S8: Multivariable analysis of risk factors of non-access-related infections requiring hospitalization in patients not in dialysis at baseline, and who do not end up with dialysis during the study period (n=118).

	Model estimates			Proportionality assumptions	
Parameters at inclusion	Hazard ratio	95% CI	p-value	χ^2	p-value
Age, years	1.03	1.01-1.07	0.08	0.116	0.734
Sex, male	0.89	0.36-2.18	0.8	0.221	0.638
Diabetes	1.20	0.54-2.71	0.65	0.364	0.546
log10 (MBL) (ng/mL)	1.11	0.65-0.90	0.71	0.028	0.867
log10 (Ficolin-1) (ng/mL)	5.43	0.36-81.06	0.22	0.027	0.869
log10 (Ficolin-2) (ng/mL)	1.28	0.16-10.20	0.82	0.225	0.635
log10 (Ficolin-3) (ng/mL)	0.60	0.30-1.18	0.14	2.056	0.152
log10 (CL-L1) (ng/mL)	0.19	0.00-630.2	0.69	0.493	0.483
log10 (C3dg) (mU/mL)	1.53	0.13-18.97	0.74	0.394	0.530
log10 (MAp44) (ng/mL)	0.70	0.01-45.0	0.87	1.639	0.2
log10 (ITIH4) (µg/mL)	0.64	0.00-366.0	0.89	0.61	0.435
Global				11.832	0.376

Figure S1: Scaled Schoenfeld residuals for C3dg, demonstrating a limited violation of the assumption of proportional hazards.

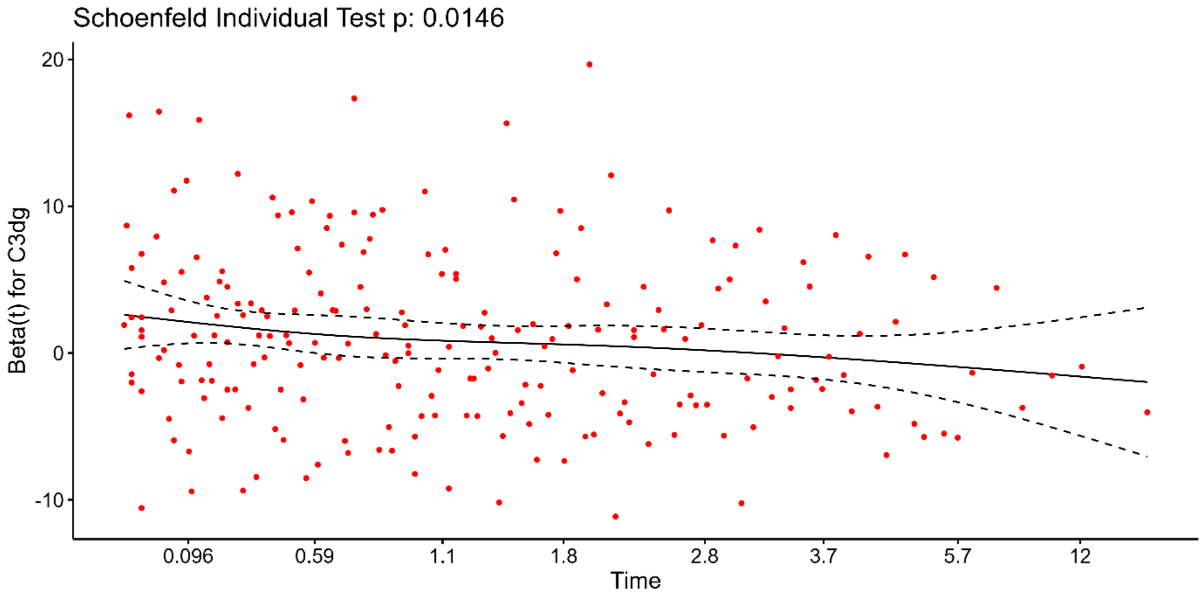

Figure S2: Variance Inflation Factors for the fully adjusted model.

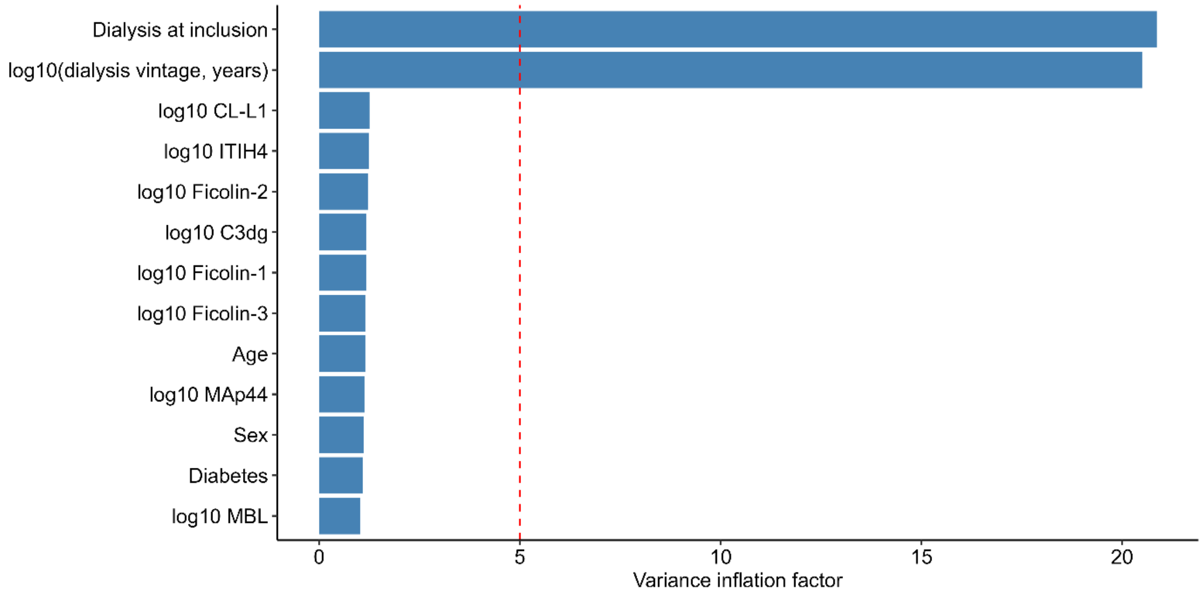

Supplement: Supplementary file 1 [file DataSheet1.pdf]
